# *Echinococcus multilocularis* in Dogs, Japan

**DOI:** 10.3201/eid1208.051241

**Published:** 2006-08

**Authors:** Yasuyuki Morishima, Hiromu Sugiyama, Kyoko Arakawa, Masanori Kawanaka

**Affiliations:** * National Institute of Infectious Diseases, Tokyo, Japan

**Keywords:** *Echinococcus multilocularis*, Parasitology, cestoda, helminths, dogs, feces, epidemiology, enzyme-linked immunosorbent assay, polymerase chain reaction, anthelminthics, letter

To the Editor: Alveolar echinococcosis in humans is endemic in Japan; however, the causal agent, Echinococcus multilocularis, has been restricted to the northernmost insular prefecture of Hokkaido, where the Tsugaru Strait acts as a natural physical barrier against migration to the mainland. Two E. multilocularis invasions into Hokkaido have occurred (*1*). The first invasion to the offshore island of Rebun in the mid-1920s was successfully controlled; however, the second invasion, supposedly in the 1940s, led to the current epidemic on the main island of Hokkaido. Both invasions were entirely or partly caused by humans who removed foxes from disease-endemic areas without taking the necessary precautions.

The finding of 19 autochthonously acquired cases of alveolar echinococcosis in prefectures other than Hokkaido (*2*) implies that the parasite exists in other areas, although the source of infection has yet to be identified. In many countries, studies of the increased spread of the parasite have traditionally focused on the contribution of foxes (*3*); however, these cases may also have been spread by domestic dogs from disease-endemic areas. Dogs are susceptible to infection with the parasite from rodents. Although the prevalence of E. multilocularis among dogs in Hokkaido is certainly lower than that in foxes (*4**–**6*), dogs can traverse considerably greater distances by various modes of transport. The number of dogs that travel from Hokkaido to other prefectures has been estimated at >12,000 per year (*7*). Although dogs may carry the parasite to remote areas, surveys of population dynamics have not been undertaken. We therefore studied the extent of E. multilocularis infection in dogs being transported by their owners from 4 ferry ports in Hokkaido (Hakodate, Muroran, Otaru, and Tomakomai) from September 2003 through October 2004.

We tested 183 fecal samples from 41 resident (in Hokkaido) and 142 nonresident dogs. We screened for the Echinococcus-specific coproantigen by using a commercial enzyme-linked immunosorbent assay kit (CHEKIT-Echinotest, Bommeli Diagnostics, Liebefeld-Bern, Switzerland) and following the manufacturer's recommendations. One dog from each group had the Echinococcus coproantigen. To confirm the specificity of the results, these 2 dogs were treated with 1 oral dose of praziquantel, 5 mg/kg. Subsequent fecal samples were subjected to coproantigen testing and specific PCR amplification according to the method of Dinkel et al. (*8*). The coproantigen test showed a significant reduction in the optical density value for both dogs, which can be interpreted as effective deworming for Echinococcus. However, different results were obtained for the PCR test, in which assays of fecal samples from the nonresident dog during the second round of nested PCR produced a single band of the expected size (Figure). Direct sequencing showed that the band was the same as bands obtained for E. multilocularis isolates from Hokkaido (GenBank accession no. AB243207). Conversely, fecal samples from the resident dog did not yield any positive PCR results.

**Figure:**
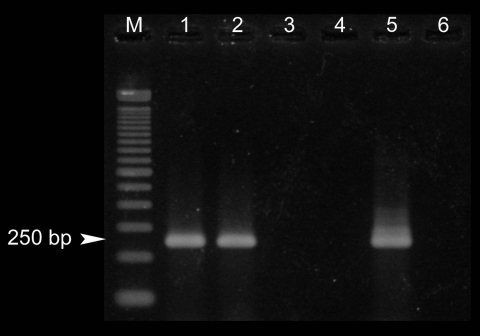
Nested PCR amplification of coproDNA from 2 coproantigen-positive dogs. Lane M, size marker (100-bp ladder); lane 1, nonresident dog (before treatment); lane 2, nonresident dog (1 day after treatment); lane 3, resident dog (before treatment); lane 4, resident dog (1 day after treatment); lane 5, positive control; lane 6, negative control. Arrowhead shows the expected band in a positive result.

The reason for the discrepancy is unclear, but it may be a false reaction in either test. Given that a reduced optical density value was obtained after administration of the taeniacidal drug, the false-positive result of the coproantigen test might have been caused by another taeniid species. Such cross-reaction has been reported previously with this test (*9*). However, no worm debris was found in the fecal samples. Alternatively, sexual maturation or low infection intensity of E. multilocularis may produce false-negative results in PCR assays (*8*). Thus, because the owner stated that the dog was allowed to roam freely and frequently preyed on rodents, this coproantigen-positive but coproDNA-negative dog was highly suspected of being infected with E. multilocularis.

Infection among wild foxes can spread to domestic dogs by way of highly contaminated rodent hosts (*10*). A nonresident dog became infected with E. multilocularis despite staying in Hokkaido for only 5 days and being permitted to roam freely for just a few hours. This finding suggests a high infection pressure of E. multilocularis to domestic dogs within the area. In addition, the increased popularity of keeping dogs as companions, greater frequency of dogs' traveling with their owners, and high prevalence in foxes from urban and rural areas in Hokkaido (*5**,**6*) all contribute to the possibility that E. multilocularis could emerge in unsuspected locations. Thus, to prevent this parasite from spreading, measures such as those used by the Pet Travel Scheme of the United Kingdom should be applied to ensure that dogs from disease-endemic areas are pretreated before entry to the main island of Japan.
